# Continuous theta-burst stimulation over the left posterior inferior frontal gyrus induced compensatory plasticity in the language network

**DOI:** 10.3389/fneur.2022.950718

**Published:** 2022-09-14

**Authors:** HyunJung An, Shahid Bashir, Eunsil Cha, Jeongeun Lee, Suk Hoon Ohn, Kwang-Ik Jung, Woo-Kyoung Yoo

**Affiliations:** ^1^Hallym Institute for Interdisciplinary Program Molecular Medicine, Hallym University College of Medicine, Chunchon, South Korea; ^2^Neuroscience Center, King Fahad Specialist Hospital Dammam, Dammam, Saudi Arabia; ^3^Department of Physical Medicine and Rehabilitation, Hallym University Sacred Heart Hospital, Anyang, South Korea

**Keywords:** inhibition, Broca area, virtual lesion, naming, non-invasive brain stimulation, repetitive transcranial magnetic stimulation

## Abstract

**Introduction:**

Continuous theta-burst stimulation (cTBS) has been used as an effective tool in inducing inhibitory aftereffect within a short time periods in the motor cortex; this has been demonstrated in the language network to a limited degree with controversial effect. In this study, we aimed to delineate the offline effect of cTBS-induced changes to the left posterior inferior frontal gyrus (pIFG) in healthy subjects using functional magnetic resonance imaging (fMRI).

**Methods:**

Twenty healthy, normal subjects (mean age: 30.84 years) were recruited. They all were right-handed and had no contra-indications for fMRI or cTBS. They were randomly assigned into the treatment group or the sham control group.

**Results:**

ANOVA showed that cTBS had a significant main effect only when the sham treatment was subtracted from the real stimulation in left superior temporal, left inferior frontal gyrus, thalamus, and right insular cortex (uncorrected *p* < 0.002). The subjects' post-cTBS condition differed significantly from their pre-cTBS condition in the left pIFG (uncorrected *p* < 0.002). There were interactions in the pIFG, bilateral superior parietal lobules, left superior temporal, left supramarginal, and left cuneus areas. The application of cTBS induced increased BOLD signals in language-related networks by stimulating the left pIFG (BA 44). This implies that inhibiting the pIFG led to increased use of language network resources.

**Conclusion:**

This study demonstrated cTBS-induced changes in the language network caused by stimulation of the left pIFG. Based on these findings, future studies on the therapeutic effects of cTBS on the right Broca's homolog area are warranted.

## Introduction

Repetitive transcranial magnetic stimulation (rTMS) is a non-invasive and relatively painless neuromodulation technique used to temporarily disrupt cortical neuronal activity. It can be used to study numerous cognitive functions and to clarify the relationship between the brain and behavior (1, 2). A novel variant form of rTMS, continuous theta burst stimulation (cTBS) (3), has various advantages, including rapid application and the ability to produce behavioral effects and induce robust neurophysiological aftereffects that are thought to involve neuronal mechanisms similar to those of long-term depression (LTD) (2–6). cTBS has an inhibitory effect that can reduce motor cortical excitability, denoted by the suppression of motor evoked potentials (MEPs), in a way that imitates the mechanisms of LTD (3, 7–11).

Functional magnetic resonance imaging (fMRI) and clinical and neurophysiological findings of non-invasive brain stimulation has been shown that the posterior inferior frontal gyrus (pIFG) plays a key role in different language functions and networks. Multiple line of evidence has been shown that the conventional rTMS on Broca's homolog area were effective for the purpose of improving aphasia after stroke (7, 12–18). Like conventional rTMS, cTBS studies mostly reported improvement of naming performance in healthy subjects (19, 20), as well as in post-stroke aphasia patients (21) by right Broca's homolog area stimulation. It is essential to have understanding how stimulation of Broca's area cause suppression or facilitation in the language network by cTBS protocol, however, it has been seldomly reported. There was one study that applied off-line cTBS over the left pIFG in healthy subjects, which suppressed activity in the left pIFG and increased activity in the right pIFG during repetition of auditory and visual words and pseudowords (22). This finding is somewhat different to note that facilitation of motor cortex using facilitatory protocol namely the intermittent TBS, which resulted reduced activity in fMRI (23), although one considers the difference of the stimulated cortex.

Combining TMS-evoked neuronal responses with cerebral hemodynamics is a valuable approach for exploring how TMS impacts neuronal activity in the targeted and remote areas that are required for specific tasks (22, 24). This approach can be used to probe the immediate effect of TMS on regional neuronal activity across the whole brain, which then makes it possible to draw inferences about the contribution of the targeted area to a specific task or function (22, 24, 25). In the current study, concurrent cTBS-fMRI was employed to investigate the effect of short bursts of high-frequency cTBS to the left pIFG on the blood-oxygen-level-dependent (BOLD) response during a picture-naming task. We aimed to assess whether real, cTBS (in contrast to sham cTBS) applied to an intact left pIFG could induce changes or modulate task-related activities in the language network during a picture-naming task (PNT).

## Methods and materials

### Participants

Twenty healthy subjects (4 females; mean age 30.2 ± 4.5 years) were recruited for this study. The subjects were randomly assigned to one of two groups: the real cTBS (treatment) group or the sham cTBS (control) group. All subjects were native Korean speakers. All were assessed as right-handed using the Edingurgh Inventory for Handedness (EHI) (26). Participants who were age between 20 and 35, didn't had any sign or history of neurological or psychiatric conditions and acute medical illness were included in this study. The exclusion criteria were use of psychotropic medication, an average use of more than three alcoholic beverages, pregnancy, previous history of head trauma or brain surgery, ferromagnetic metal in the head, implanted cardiac pacemaker or neurostimulator. All participants provided written informed consent before participating but after the study procedure was fully explained to them. The study was approved by the local research ethics committee and conducted in accordance with the Declaration of Helsinki.

### Experimental setup and design

The stimulation set-up consisted of a magnetic stimulator STM 9000 (ATES MEDICA Device, Italy) that administered single-pulse TMS and cTBS. A figure-8 coil was placed tangentially over the subject's left primary motor cortex with the handle pointing at a 45° angle posterolaterally. To measure MEP, a surface electromyography (EMG) was performed using pre-gelled, disposable Ag/AgCl electrodes. The active electrode was placed on the contralateral first dorsal interosseous (FDI) muscle, the reference electrode on the metacarpophalangeal joint, and the ground electrode on the wrist. The EMG signal was acquired at 3 kHz, filtered (10–500 Hz), amplified, and stored for offline analysis.

During the procedure, the participants sat in a comfortable chair with a headrest; their hands rested on their laps. They were monitored for drowsiness and asked to keep their eyes open during TMS. All participants wore earplugs to protect them from possible acoustic trauma due to the noise from the discharge of the TMS coil.

**Figure 1:**
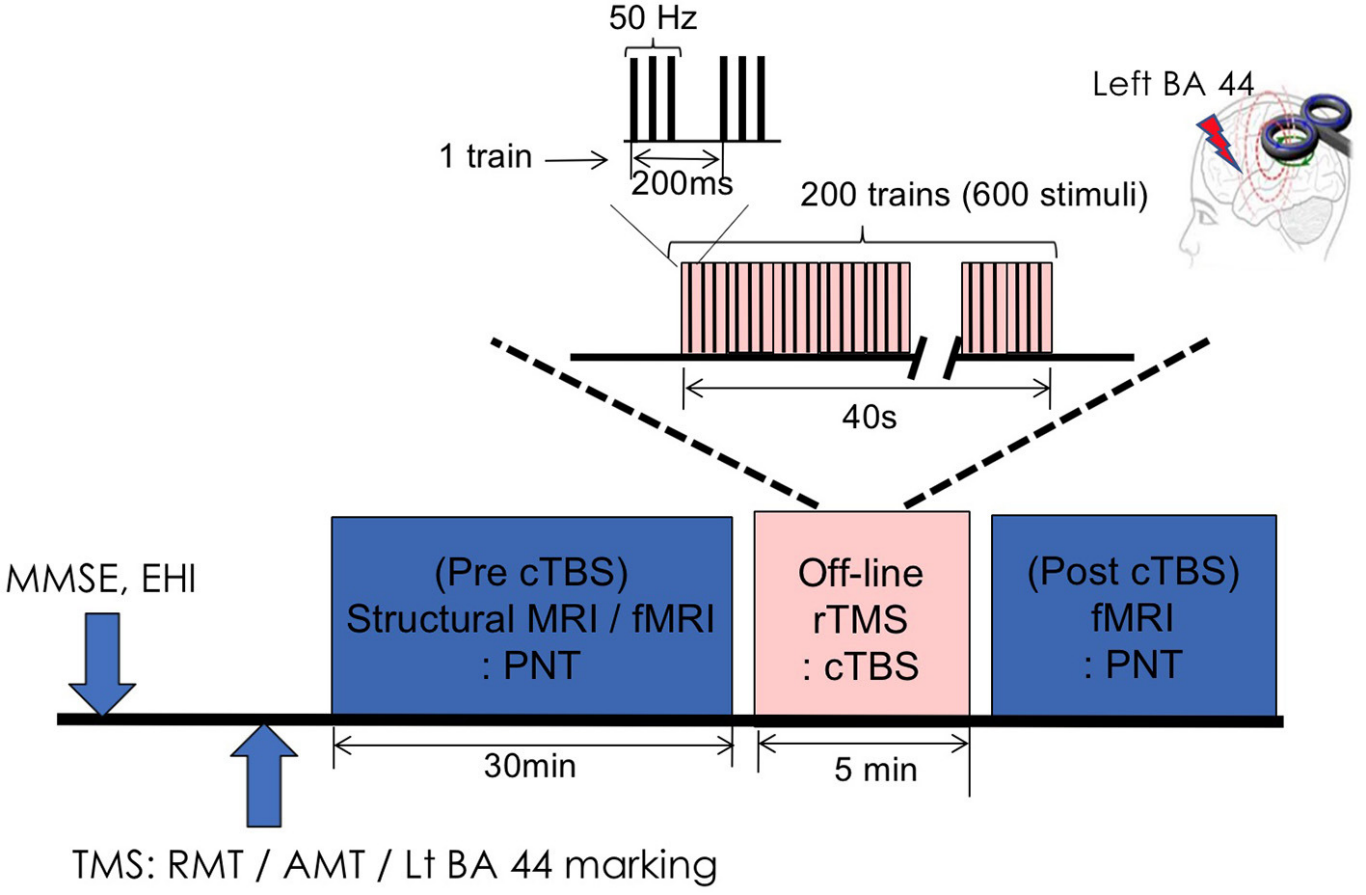
Experimental design. MMSE, Mini Mental Status Examination; EHQ, Edinburgh Handedness Inventory; TMS, transcranial magnetic stimulation; RMT, resting motor threshold; AMT, active motor threshold; BA, Brodmann area; rTMS, repetitive transcranial magnetic stimulation; cTBS, continuous theta-burst stimulation; PNT, picture naming task.

All participants underwent baseline tests (MMSE, EHI) and imaging study (structural MRI and fMRI) followed by baseline TMS measurement [resting motor threshold (RMT) and active motor threshold (AMT)] (Figure 1). Pre-cTBS fMRI was done using PNT. Then participants moved to another room and received cTBS on left pars opercularis for 40 s. Right after cTBS procedure, the participants undergone post-cTBS fMRI using same task with different set of pictures (Figure 1).

### Task and FMRI procedure

The participants completed a PNT that used pictures of simple, white-on-black line drawings (27). Thirty pictures were selected for the fMRI overt naming task; the pictures were controlled for difficulty and frequency. We prepared five different picture sets of 100 images with a moderate level of use frequency and word complexity. Different pictures were used for the pre- and post-fMRI tasks. The pictures were presented on a screen that was reflected by a mirror placed in front of the participant. Each participant performed three scanning fMRI runs with a block design of ~5 min for each. Within each run, there were five task blocks. Each block consisted of 30 s of rest and 30 s for each task (PNT). Within each task block, each trial began with 200 ms fixation; the picture was then presented for 100 ms. This was followed by a 200-ms response period during which participants were asked to name the presented picture as quickly and accurately as possible. We measured the correctness of the responses by recording participants' responses through a speaker outside the scanner. For the behavioral task, two investigators independently count the behavioral response. We only counted both investigators' positive answers.

#### FMRI acquisition

FMRI was acquired using a single-shot gradient echo planar imaging (EPI) sequence. The vast majority of fMRI performed at field strengths of 3T and below uses single-shot EPI, in which the k-space representation of an excited slice is read out in a single extended echo train. These extended readout periods make EPI sequences very efficient in terms of SNR per unit time, and thus highly sensitive to functional activation (28). The scanning parameters were as follows: 190 EPI volumes per block; repetition time (TR)/echo time (TE) = 2,000/35 ms; flip angle (FA) = 90°; field of view (FoV) = 240 mm; matrix = 64 × 64; resolution = 3 × 3 × 3 mm^3^; and 35 slices. A high-resolution structural T1-weighted image was obtained using a magnetization-prepared rapid acquisition gradient echo (MP-RAGE) sequence with the following scanning parameters: TR/TE = 8.1/2.3 ms; thickness = 1 mm; FoV = 256 mm; FA = 90°; matrix size = 256 × 256, and resolution = 1 × 1 × 1 mm^3^.

#### FMRI analysis

The data were analyzed using the Statistical Parametric Mapping software (SPM8, Wellcome Department of Imaging Neuroscience, London, UK) with Matlab version 2009a (Mathworks, Natick, MA). Prior to analysis, all images were preprocessed. The images were realigned to the mean functional image in the time series using mean frame-wise displacement, co-registered with each individual's structural image, spatially normalized to the MNI space, and spatially smoothed using a Gaussian kernel (8 mm, full width at half maximum). Poor-quality scans due to excessive head movement (≥3 mm) were excluded from the analysis. One case in sham condition showed excessive head movement, we excluded it.

For the subject-level analysis of the fMRI data, a general linear model (GLM) was used to calculate individual contrasts. The design matrix consisted of the PNT and rest periods. Motion parameters were included in the design as regressor variables of no interest to exclude variance related to head movement. Contrast maps for each condition were created for all participants.

For the group-level analysis, a full-factorial ANOVA with two independent variables (pre- and post-cTBS) and two dependent variables (real and sham cTBS) was conducted. The statistical contrast maps were thresholded. The height threshold was *p* < 0.002 (uncorrected) at the voxel level, and the extent threshold was 10 voxels with a false-discovery rate (FDR) correction at the cluster level of *p* < 0.05.

#### ROIs and analysis

Since we targeted the left pars opercularis gyrus (BA 44) for stimulation, changes to the network related to the left BA 44 were our main interest. To explore the effects of real and sham cTBS pre- and post-stimulation, we determined functionally defined regions of interest (ROIs) (left BA 45 [−48, 24, −10]; left BA 44 [−38, 16, 50]; right BA 45 [52, 22, −10]; right BA 44 [44, 8, 54]) using the Automated Anatomical Labeling (AAL-90) atlas (29). We then extracted the contrast estimate for each subject individually.

### TMS stimulation

TMS was conducted in a separate, quiet room near the MRI center. This study was designed to explore the offline effect of cTBS, so we conducted an fMRI on each subject before and immediately after cTBS was applied to Broca's area.

The RMT was obtained over M1, where the lowest stimulus intensity evoked TMS-induced motor evoked potentials (MEPs) of at least 50 μV in the target muscle in five out of 10 consecutive trials. The TMS sessions were conducted in accordance with the published safety guidelines (30, 31). The targeted area for the stimulation (left pars opercularis gyrus) was identified using MRIcro software after a 3D T1 magnetic resonance image (MRI) was obtained with a surface marker located near both pIFG (BA 44). An offline-cTBS was administered with 40-s trains of 3 bursts at 50 Hz pulses, interleaved by 200 ms, for a total of 600 pulses. The mean stimulator output was delivered at 90% of the individual AMT. For each session, AMT was calculated for the left first dorsal interosseous and defined as the minimum stimulator output intensity required to produce motor evoked potentials (MEP) of at least 200 μV at 20% of maximum muscle contraction (3).

Sham group received stimulation by tilting the stimulator coil 45 degree so that subjects feel the pressure on the scalp along with a same auditory input.

### Statistical analysis

For the subject-level analysis of the fMRI data, a general linear model (GLM) was used to calculate individual contrasts. For the group-level analysis, a full-factorial ANOVA with two independent variables (pre- and post-cTBS) and two dependent variables (real and sham cTBS) was conducted. The height threshold was *p* < 0.002 (uncorrected) at the voxel level, and the extent threshold was 10 voxels with a false-discovery rate (FDR) correction at the cluster level of *p* < 0.05. The accuracy of PNT in fMRI session was compared between real and sham group using independent *t*-test.

## Results

### fMRI whole-brain analysis

**Figure 2:**
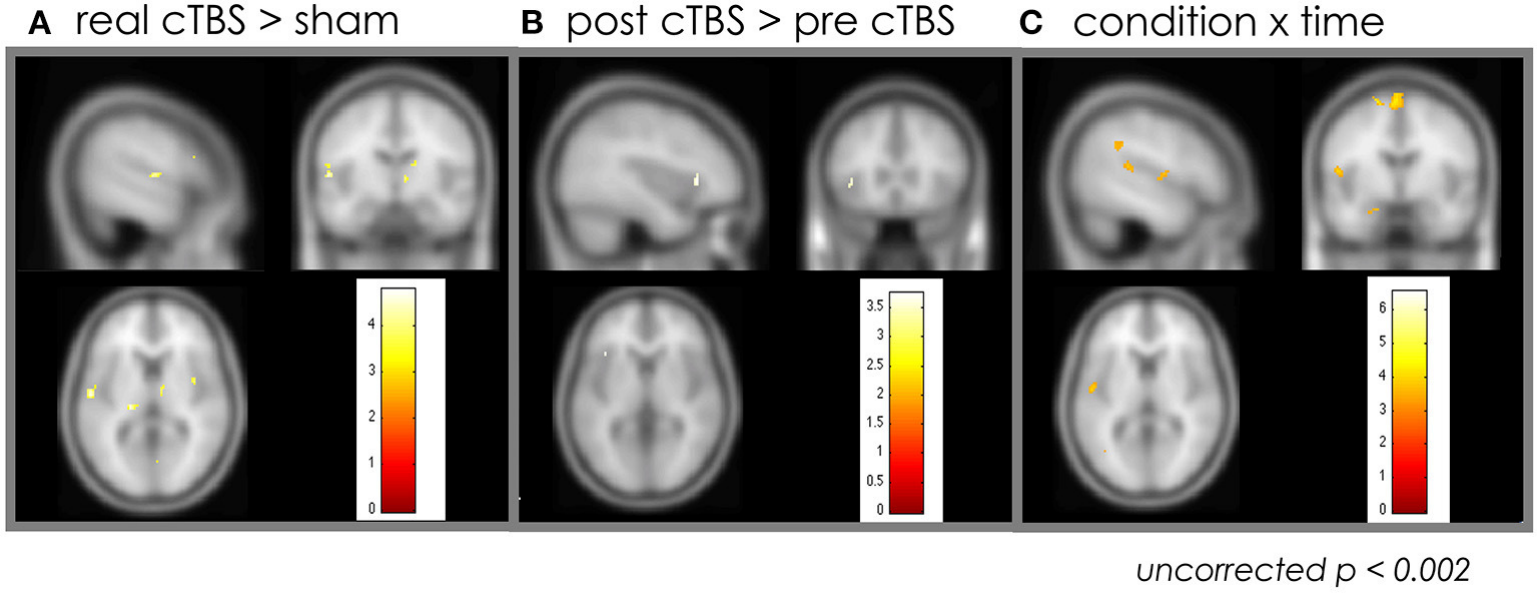
Effect of the cTBS: whole brain analysis. **(A)** Increased activation in the right insula, right pIFG, left superior temporal gyrus, left middle temporal gyrus, and left middle frontal area in the real cTBS group compared to sham group. **(B)** The post-cTBS MRI showed significantly increased activation only in the left pIFG compared to pre-cTBS. **(C)** A significant interaction effect between condition and time was observed in the left pIFG, the left superior temporal gyrus, both superior parietal lobules, the left angular gyrus, and the left cuneus. cTBS, continuous theta-burst stimulation; pIFG, posterior inferior frontal gyrus; MRI, magnetic resonance imaging.

ANOVA showed increased activation in the right insula, right pIFG, left superior temporal gyrus, left middle temporal gyrus, and left middle frontal area in the real cTBS group (uncorrected *p* < 0.002) (Figure 2A); there was no significant increase in activation in the sham cTBS group. The post-cTBS MRI showed significantly increased activation only in the left pIFG (uncorrected *p* < 0.002) (Figure 2B). There was no significant activation in the pre-cTBS compared to post-cTBS condition. A significant interaction effect between condition and time was observed in the left pIFG, the left superior temporal gyrus, both superior parietal lobules, the left angular gyrus, and the left cuneus; in all these areas, activation increased significantly post-cTBS in the treatment group (uncorrected *p* < 0.002) (Table 1) (Figure 2C).

**Table 1:** The area of increased activation by the interaction effect between condition and time.

**Region**	**BA**	**x**	**y**	**z**	***P*** **-value**
Left posterior inferior frontal gyrus	44	−51	7	9	<0.001
superior temporal gyrus	22	−52	−34	15	<0.001
angular gyrus	39	−44	−46	33	<0.001
cuneus	19	−13	−74	28	<0.001
superior parietal lobule	7	−6	−35	66	<0.001
Right superior parietal lobule	7	13	−64	52	<0.001

BA, Brodmann area.

### fMRI ROI analysis

**Figure 3:**
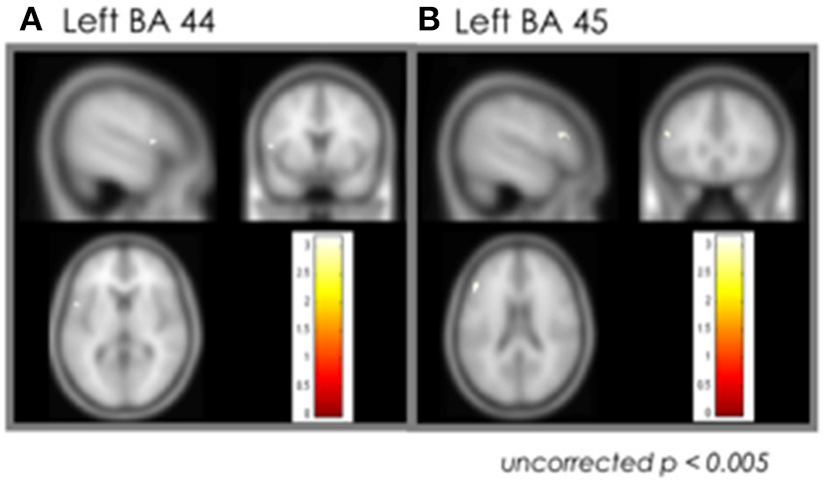
Effect of the cTBS: ROI analysis. **(A)** Left BA 44. **(B)** Left BA 45. cTBS, continuous theta-burst stimulation; BA, Brodmann area.

The ROI analysis focusing on the language areas showed increased activation only in left BA 45 and 44 areas of the post cTBS stimulation condition (uncorrected *p* < 0.005) (Figure 3).

### Behavioral results

All subjects in both groups achieved 100% accuracy on the picture-naming task; no behavioral differences between the two groups were observed.

## Discussion

The current work aimed to investigate the impact of cTBS applied to the left pIFG on the hemodynamic response during a PNT. We found that cTBS had a significant effect on the left pIFG, left superior temporal gyrus, bilateral superior parietal lobule, left angular gyrus, and left cuneus. These findings suggest two possible interpretations. First, offline-cTBS may have increased neuronal activity in the stimulated area (left pIFG) and in other brain regions within the language network during the PNT. This would indicate that the inhibitory effect of cTBS led to the recruitment of additional resources to achieve similar performance (12–16). Second, the inhibitory aftereffect of cTBS may have modulated intrahemispheric cortical connectivity; this explanation aligns with recent previous work done using TMS-EEG (32).

Administering cTBS at 80% of AMT to the left pIFG has been reported to lead to no change in behavioral outcomes on a speech repetition task (33). Other studies have found that cTBS induced a significant decrease in pIFG and M1 effective connectivity during a listening task (34), suppressed activity in the left pIFG and increased activity in the right pIFG during repetition of words and pseudowords (22) and led to changes in interhemispheric and intrahemispheric TMS-evoked synchrony as measured by electroencephalogram (EEG) (32). In this study, since we used a higher intensity (90% of AMT), it is not surprising that we observed changes in the stimulated area and in the related network. It is interesting, however, that activation in the target area increased rather than decreased; this contrasts with previous reports (22). The different tasks used in this and previous studies (picture naming vs. contrasting words and pseudowords repetition) and differences in stimulation intensity (90% vs. 80% of AMT) cannot be excluded as explanations for this contradiction. Moreover, since the basic principle of activation in fMRI is that activation depends on the potential effectiveness of the resource, this finding could be interpreted to indicate decreased effectiveness in the stimulated hemisphere since more resources were recruited to perform the task (we observed no additional activation in the right inferior frontal area). This explanation would align with previous studies of iTBS stimulation to the motor cortex (23).

The language related other nodes were increased after cTBS that includes left posterior part of superior temporal gyrus and left angular gyrus. Among left superior temporal gyrus, posterior and ventral to Heschel's gyrus portion has been reported to have a phonological priming effect, which is lateralized speech selective response (35). The angular gyrus is one of the node of semantic retrieval network, which is widely distributed in a left-lateralized network including in the inferior temporal gyrus, anterior fusiform, hippocampus, pars orbitalis, superior and middle temporal gyri, and the right cerebellum (36). Especially, left angular gyrus is related to word comprehension in both speaking and writing (37).

Together with both bilateral superior parietal lobule and cuneus involved in visuospatial attention, above language network would be used to increase activity to compensate inhibition induced by cTBS by use of phonological and semantic resources as well as by increasing signal to noise ration with attention.

PNT is a sequential process of relation of visual recognition of picture by semantic network into lexical network, which represents concepts' names leading to speech production (38). In this semantic retrieval process, semantically related words showed reduced activity in the left pars orbitalis than phonologically related words (39). In this study, the significant interaction effect was located at BA 44 [−51, 7, 9]. This region has been reported to have relation to articulating planning (40, 41). As this area is also involved in working memory increased activity in the real cTBS condition might have related to difficult speech comprehension that reflects top-down predictions about the possible articulatory codes associated with the words (42, 43).

One main strength of this work is that it combined rTMS with fMRI to probe the impact of short bursts of high-frequency stimulation (cTBS) to the left pIFG on the BOLD hemodynamic response during a language task. However, this study has some limitations. First, caution must be used when interpreting fMRI in general, because of the small number of participants. Second, this study obtained limited behavioral data, which is a major drawback. Behavioral data (i.e., reaction time on a millisecond scale) could not be recorded due to background noise, so only the correctness of the responses was acquired. Third, the criteria of head motion that we excluded from the analysis might be too loose, which couldn't exclude to have artifacts instead of real activations, although we included the estimated motion parameters in the design as regressor of no interest in the first level GLM, which can reduce the sensitivity to detect noise as well as activations. Finally, the fMRI data were associated only with the onsets of correct trials in the PNT. Analyzing behavioral data and correlating them with the fMRI data would expand the current findings by relating them to behavioral measures. Future work should employ online and offline TMS coupled with fMRI data to explore the effects of TMS on electrophysiological measures (i.e., MEP amplitude), behavioral measures with more sensitive behavioral task to reduce the ceiling effect (i.e., reaction time and error rate) and on regional brain hemodynamic neuronal activity.

## Conclusion

This study demonstrated cTBS-induced inhibition in the language network caused by stimulation of the left pIFG, which induced increased use of resources of language and attention network to maintain same performance in PNT.

## Data availability statement

The original contributions presented in the study are included in the article/supplementary material, further inquiries can be directed to the corresponding author/s.

## Ethics statement

The studies involving human participants were reviewed and approved by Ethics Committee of Hallym University Sacred Heart Hospital. The patients/participants provided their written informed consent to participate in this study.

## Author contributions

W-KY and HA: design study and analysis the data. W-KY, HA, and EC: collect the data. W-KY, SB, EC, JL, SO, and K-IJ wrote the main manuscript text. All authors reviewed the manuscript.

## Funding

This research was supported by the National Research Foundation of Korea, Basic Research Promotion Fund (NRF-2013R1A1A2012562) and Hallym University Research Fund.

## Conflict of interest

The authors declare that the research was conducted in the absence of any commercial or financial relationships that could be construed as a potential conflict of interest.

## Publisher's note

All claims expressed in this article are solely those of the authors and do not necessarily represent those of their affiliated organizations, or those of the publisher, the editors and the reviewers. Any product that may be evaluated in this article, or claim that may be made by its manufacturer, is not guaranteed or endorsed by the publisher.
